# Gadolinium and Polythiophene Functionalized Polyurea Polymer Dots as Fluoro-Magnetic Nanoprobes

**DOI:** 10.3390/nano12040642

**Published:** 2022-02-14

**Authors:** Soner Karabacak, Alagappan Palaniappan, Tsang Siu Hon Tony, Teo Hang Tong Edwin, Balázs Gulyás, Parasuraman Padmanabhan, Ümit Hakan Yildiz

**Affiliations:** 1 Department of Chemistry, Izmir Institute of Technology, Urla 35430, Izmir, Turkey; sonerkarabacak@iyte.edu.tr; 2 School of Materials Science and Engineering, Nanyang Technological University, Singapore 639798, Singapore; alps@ntu.edu.sg (A.P.); e-e-htteo@ntu.edu.sg (T.H.T.E.); 3 Temasek Laboratories@NTU, 50 Nanyang Avenue, Singapore 639798, Singapore; shtsang@ntu.edu.sg; 4 School of Electrical and Electronic Engineering, Nanyang Technological University, Singapore 639798, Singapore; 5 Lee Kong Chian School of Medicine, Nanyang Technological University, Singapore 636921, Singapore; balazs.gulyas@ntu.edu.sg; 6 Cognitive Neuroimaging Centre, Nanyang Technological University, 59 Nanyang Drive, Singapore 636921, Singapore; 7 Department of Clinical Neuroscience, Karolinska Institute, 17176 Stockholm, Sweden; 8 Department of Polymer Science and Engineering, Izmir Institute of Technology, Urla 35430, Izmir, Turkey; 9 Denge Kimya, Velimese Industrial Region St. Ergene, Corlu 59860, Tekirdag, Turkey

**Keywords:** dual-functional reporters, PTAA, polymer dots, polyurea Pdots, mini-emulsion polymerization, magnetic manipulation, fluorescent reporters, bioimaging

## Abstract

A rapid and one-pot synthesis of poly 3-thiopheneacetic acid (PTAA) functionalized polyurea polymer dots (Pdots) using polyethyleneimine and isophorone diisocyanate is reported. The one-pot mini-emulsion polymerization technique yielded Pdots with an average diameter of ~20 nm. The size, shape, and concentration of the surface functional groups could be controlled by altering the synthesis parameters such as ultrasonication time, concentration of the surfactant, and crosslinking agent, and the types of isocyanates utilized for the synthesis. Colloidal properties of Pdots were characterized using dynamic light scattering and zeta potential measurements. The spherical geometry of Pdots was confirmed by scanning electron microscopy. The Pdots were post-functionalized by 1,4,7,10 tetraazacyclododecane-1,4,7,10-tetraacetic acid for chelating gadolinium nanoparticles (Gd^3+^) that provide magnetic properties to the Pdots. Thus, the synthesized Pdots possess fluorescent and magnetic properties, imparted by PTAA and Gd^3+^, respectively. Fluorescence spectroscopy and microscopy revealed that the synthesized dual-functional Gd^3+^-Pdots exhibited detectable fluorescent signals even at lower concentrations. Magnetic levitation experiments indicated that the Gd^3+^-Pdots could be easily manipulated via an external magnetic field. These findings illustrate that the dua- functional Gd^3+^-Pdots could be potentially utilized as fluorescent reporters that can be magnetically manipulated for bioimaging applications.

## 1. Introduction

A wide range of probes for magnetic resonance imaging [1], X-ray computed tomography [2,3,4], positron emission tomography [5,6], and fluorescence imaging [7,8] have been explored to facilitate therapy and diagnosis. Over the recent years, emphasis has been laid on the development of probes with more than one functionality. Polymer dots, a class of fluorescence imaging probes with high fluorescence intensity, photostability, and high biocompatibility are one of the ideal candidates for the development of multi-functional probes [9,10]. Polymer dots based nanoprobes have revolutionized common practice in bioimaging [11,12,13,14,15,16], diagnostics, and therapeutic applications [17,18]. The major advantages of polymer dot nanoprobes are their superior photophysical properties and post-functionalization capabilities. Therefore, polymer dots enable imaging of a wide range of samples from single cell to more complex tissues and organs.

The current reports on polymer dots exhibit their potential for in-vitro and in-vivo imaging and diagnosis [19,20,21]. Single chain polymer dots in reduced diameter (<10 nm) have shown improved quantum yield, photostability, and colloidal stability [21,22,23,24,25,26,27,28] as compared to larger diameter polymer dots. Recently, Ozenler et al. demonstrated the use of single-chain polymer dots for differentiation of cancer and healthy cells in co-culture medium [29]. Though single chain polymer dots are expected to show unambiguous advantages in imaging, post-functionalization possibilities are still limited due to the small surface area. Considering the current requirements in clinical practice, bioimaging probes capable of rapid functionalization, bioconjugation, and multimodal imaging are of high priority [30]. Further, the multi-functional probes should be biocompatible and facilitate high-resolution bioimaging. Recently, dual-functional fluoro-magnetic reporters have gained significant research attention as they enable visualization of several biological processes [31].

In this study, a facile one-pot approach [32] for the synthesis of dual-functional fluoro-magnetic polymer dots reporters is described. Poly 3-thiopheneacetic acid (PTAA) [33] fluorescent reporters are added to a mixture of polyethyleneimine (PEI), isophorone diisocyanate (IPDI) to yield PTAA functionalized polyurea polymer dots (Pdots) via a one -pot mini-emulsion technique. The adopted mini-emulsion technique enables precise control of size, shape, and concentration of the surface functional groups by varying the parameters such as ultrasonication time, concentration of the surfactant, and crosslinking agent, and the types of isocyanates utilized for the synthesis. The Pdots were post-functionalized by 1,4,7,10 tetraazacyclododecane-1,4,7,10-tetraacetic acid (DOTA) for chelating gadolinium nanoparticles (Gd^3+^) that impart magnetic properties to the Pdots. Hence, the PTAA and Gd^3+^ serve as dual-functional fluorescent and magnetic reporters, respectively, for multimodal bioimaging. The optical and magnetic properties of the Gd^3+^-Pdots characterized using fluorescence spectroscopy and a magnetic levitation system, reveal detectable fluorescent signals even at lower concentrations and facile manipulation by an external magnetic field. Thus, the proposed one-pot mini-emulsion synthesis approach could be utilized to synthesize dual-functional Gd^3+^-Pdots for high-resolution multimodal imaging of various biological samples.

## 2. Materials and Methods

**Scheme 1:**
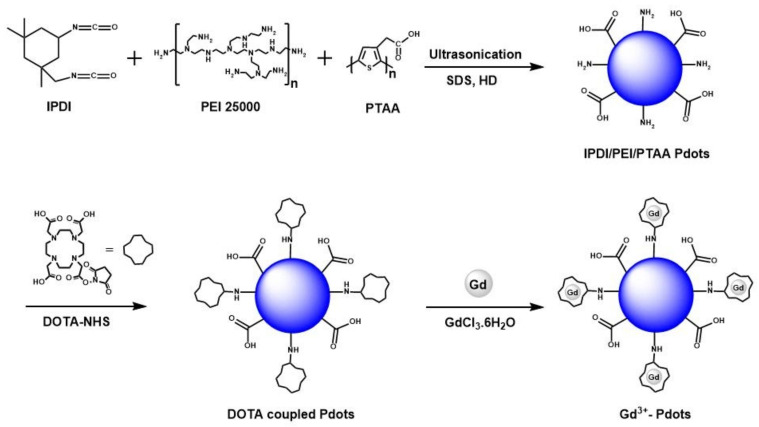
Schematic representation of the preparation of Gd^3+^-Pdots dual-functional reporters (Overall yield is 26.6% for Gd^3+^-Pdots dual-functional reporters).

Dual-functional Gd^3+^-Pdots were synthesized as shown in Scheme 1. Pdots were prepared via a one-pot mini-emulsion technique. IPDI (0.21 mL, 1 mmol), PEI 25,000 (1 g, 0.04 mmol), PTAA (1.25 mg in 250 µL water, pH: 9), hexadecane (114 µL, 0.387 mmol), and sodium dodecyl sulfate (SDS) (84 mg, 0.294 mmol) in 10 mL DI water were added to a 50 mL round-bottom flask and stirred for 1 h at room temperature. The pre-emulsion solution was then ultrasonicated for 2 min. After ultrasonication, the mixture was added to a 50 mL round-bottom flask, and the reaction mixture was refluxed at 60 °C for 4 h and then cooled to room temperature to yield the Pdots solution.

DOTA-NHS (10 mg, 20 µmol) in 100 µL phosphate-buffered saline (PBS) solution was added to 100 µL of Pdots solution with a magnetic stirrer and stirred for 1 day. Then, gadolinium (III) chloride hexahydrate (GdCl_3_∙6H_2_O) (75 mg, 0.2 mmol) was added to 2 mL citric acid monohydrate solution (230 mg, 0.6 mmol) and stirred for 3 days, followed by purification using a 12–14 kDa dialysis tube and to remove excess Gd^3+^ in solution. Five cycles of dialysis (each cycle for 24 h) were performed against a 0.05 M citrate solution (200 mL, pH: 7.4) to yield the dual-functional Gd^3+^-Pdots reporters. All the pH adjustments during the synthesis of Gd^3+^-Pdots were performed using NaOH and HCl.

## 3. Results and Discussion

Anionic PTAA was synthesized and characterized using NMR, UV-visible spectroscopy, and fluorescence spectroscopy to synthesize the Pdots fluorescence reporters. Firstly, poly 3-thiophene methyl acetate (PTMA) was characterized using ^1^H NMR (Figure S1, 400 MHz, CDCl_3_, δ, ppm) that showed associated peaks at 7.30–7.00 (proton of thiophene ring, m, 1H), 3.70 (s, thiophene ring, 2H), and 3.60 (s, methyl, 3H). Then, FTIR-ATR spectroscopy was performed to compare PTMA and PTAA, as shown in Figure S2. It could be observed from Figure S2 that aromatic ester (C-O) functional group was observed in PTMA spectrum at 1310 cm^−1^ and 1270 cm^−1^ but not in the PTAA spectrum. This difference plays a key role in the characterization of PTAA. The most significant peak of the spectrum is the broad O-H peak observed in the 3400–2400 cm^−1^ range. Moreover, the carbonyl peak at 1700 cm^−1^ reveals the existence of the carboxyl group. The absorption peak at 3180–2980 cm^−1^ range refers to the C–H bond on the ring of thiophene, and the aliphatic C-H bond is observed at 2980–2780 cm^−1^ range.

**Figure 1:**
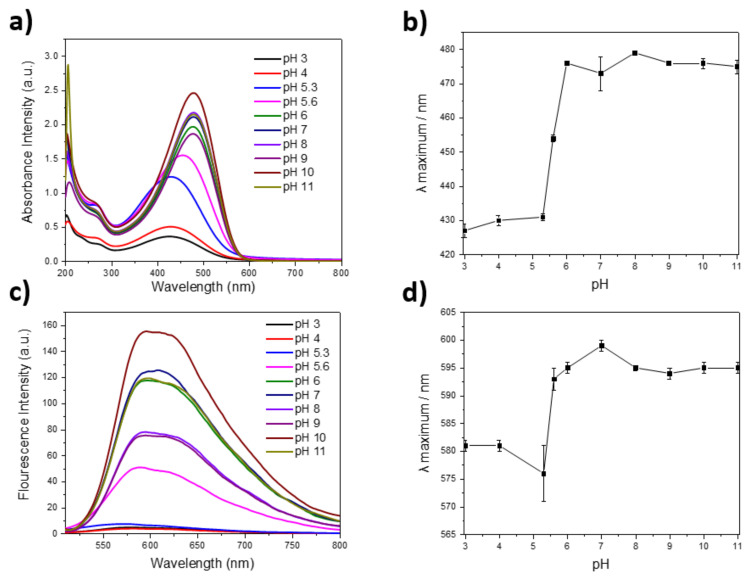
Optical characterization of PTAA (**a**) UV-visible spectrum of PTAA aqueous solution with 1.0 M NaCl concentrations at pH from 3 to 11 (**b**) UV-visible spectrum maximum wavelengths of PTAA at different pH (**c**) fluorescence spectrum of PTAA aqueous solution with 1.0 M NaCl concentrations at pH from 3 to 11 (**d**) fluorescence spectrum maximum wavelengths of PTAA at in different pH.

UV-visible and fluorescence spectroscopy was carried out for PTAA at various pH to comprehend the pH sensitivity. The pH was adjusted between pH 3 and 11 at room temperature. When the PTAA solution was prepared, 1 M NaCl was used for all samples to provide a constant ionic strength. As observed from Figure 1a, the solubility of PTAA increases with pH, as revealed by the increase in absorbance intensity at maximum wavelength. A significant increase in UV maximum wavelength (λ maximum) is observed at pH range 5–6 as shown in Figure 1b, which demonstrates the conformational changes of PTAA. In addition, the fluorescence intensity decreases with pH (Figure 1c), as examined using 1.0 M NaCl solutions. As shown in Figure 1d, the maximum wavelength of the emission spectrum shows a significant increase at the pH range 5–6. These observations further indicate that PTAA exhibits good emission intensities (Figure 1c) around physiological pH ranges, making them an ideal candidate for the development of fluorescent reporters for bioimaging.

**Figure 2:**
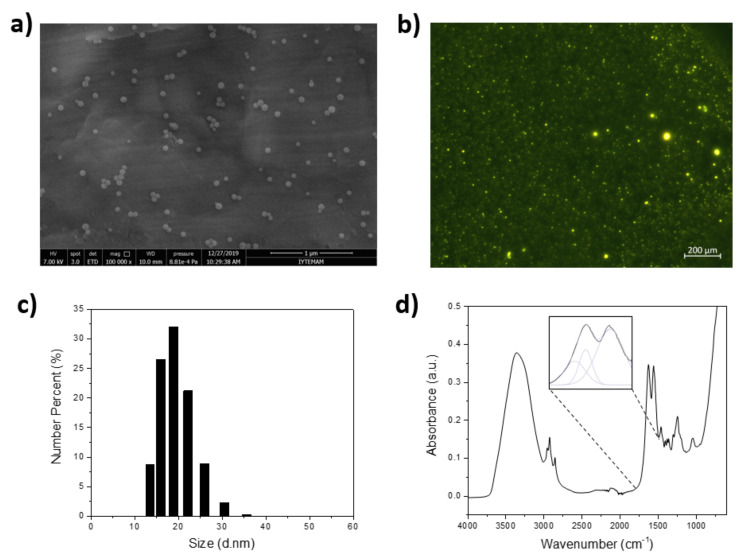
(**a**) SEM image of Pdots. (**b**) FM image of Pdots (**c**) particle size analysis of Pdots (**d**) FTIR-ATR spectrum of Pdots.

PTAA was then utilized as the fluorescence reporter to synthesize the Pdots using the one-pot mini-emulsion technique. SEM imaging of 1000 fold diluted Pdots reveals their spherical morphology (Figure 2a). Figure 2b shows fluorescence microscopy (FM) imaging of Pdots confirming spherical geometry in solution. The particle size of Pdots measured using DLS analysis indicates an average hydrodynamic radius of ~20 nm, as shown in Figure 2c, which agrees with SEM imaging. Pdots analyzed via FTIR-ATR spectroscopy (Figure 2d) yielded a broad band spanning from 3600 to 3000 cm^−1^, which is attributed to the carboxylic acid -OH groups in the PTAA structure. The peaks associated with the carboxylic acid (C=O stretching mode of carboxyl group) is observed at 1715 cm^−1^ (deconvoluted spectrum is shown in dotted lines). The NH_2_ deformation mode peak is observed at 1636 cm^−1^, indicating that the surface of Pdots contains both COOH and NH_2_ groups. These characterization results illustrate successful synthesis of fluorescent Pdots.

**Figure 3:**
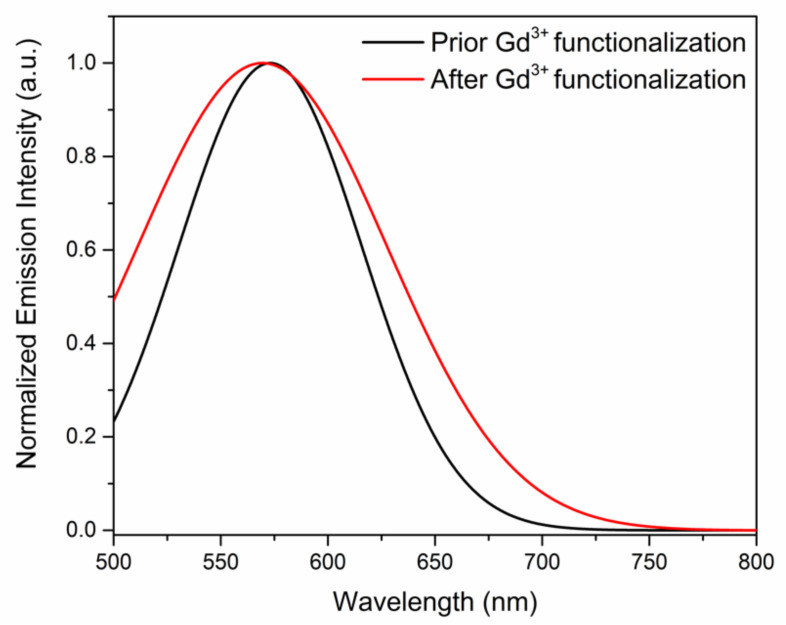
Emission spectrum of Pdots prior and after Gd^3+^ functionalization, respectively.

Upon successful characterization of Pdots, a post-functionalization process was carried out to conjugate Gd^3+^ to impart magnetic properties to the Pdots. The Pdots prior and after Gd^3+^ functionalization were analyzed by fluorescence spectroscopy shown in Figure 3 (excited at 475 nm), which yielded a broad emission spectrum with a peak maximum at 570 nm. The fluorescence intensity of Pdots and Gd^3+^ -Pdots were found to be identical, which ascertains negligible interference of Gd^3+^ functionalization on the optical properties of PTAA. The slight shift in the emission maximum could be attributed to the change in the chemical environment of Pdots by Gd^3+^ cations. The colloidal properties of Pdots and Gd^3+^-Pdots were then characterized by Zeta potential measurements, which further confirmed Gd^3+^ post-functionalization process. Prior to the functionalization of DOTA and Gd^3+^ chelation, higher concentrations of COO^−^ groups were available on Pdots, whereas the COO^−^ groups were consumed after functionalization. The zeta potential of 11.8 and 6.10 mV was measured for Pdots prior and after Gd^3+^ functionalization. The change in zeta potential was attributed to the reaction between primary amine groups and DOTA, thus, the decrement shows the binding of DOTA to the Pdots. The mobility of dual-functional reporters decreases with the functionalization of Gd^3+^ on Pdots because the size of the dual-functional reporters is larger than Pdots. Additionally, no significant difference was observed for the conductivity prior and after Gd^3+^ functionalization, as shown in Table 1, indicating that Gd^3+^ -Pdots solution does not contain free Gd^3+^ ions.

**Figure 4:**
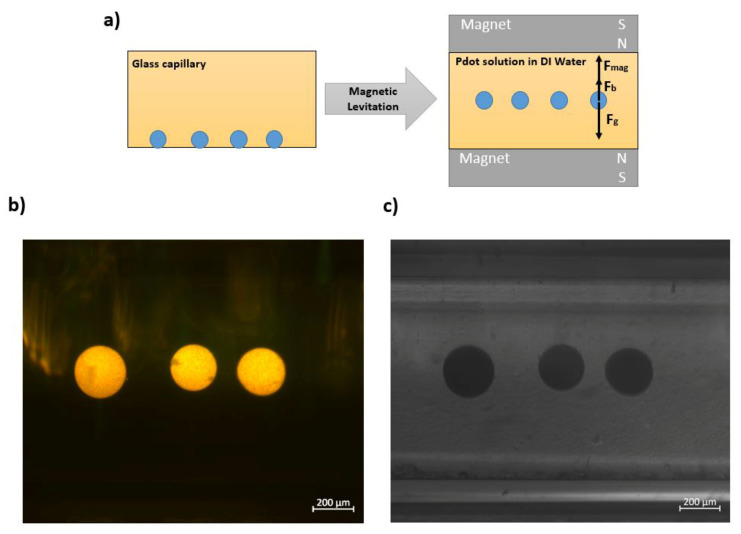
(**a**) Schematic illustration of the magnetic levitation system (**b**) fluorescence and (**c**) optical images of the polystyrene beads in the presence of magnets.

The Gd^3+^ chelation provides magnetic properties to Pdots, enabling magnetic manipulation of as well as magnetic levitation of Pdots. As shown in Figure 4a, the customized magnetic levitation system is fabricated using a glass capillary filled with Gd^3+^-Pdots solution and sandwiched between two magnets, where the same poles are positioned against each other. Polystyrene beads are utilized to illustrate the magnetic properties of Gd^3+^-Pdots. As schematically illustrated in Figure 4a, in the absence of magnets, the polystyrene beads rest at the bottom of the glass capillary since they have a higher density than Gd^3+^-Pdots solution filled in capillary, whereas they levitate at a certain height from the bottom of the glass capillary in the presence of the magnets. Figure 4b,c shows the fluorescence and optical microscope images of the levitating polystyrene beads (polystyrene beads with a density = 1.08 g/mL) in the presence of a magnet. The levitation of polystyrene beads is due to the effect of the paramagnetic behavior of Gd^3+^-Pdots, where F_buoyancy_+ F_magnetic_ > F_gravitation_.

**Figure 5:**
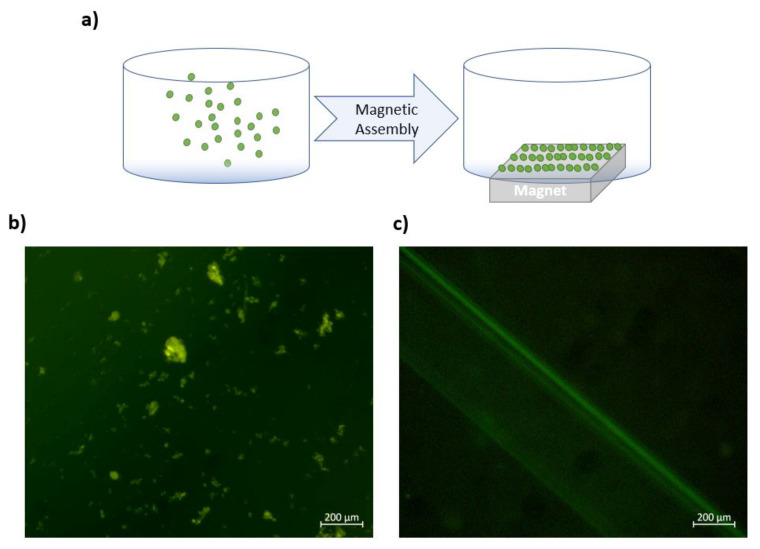
(**a**) Schematic representation of assembly of Gd^3+^-Pdots on the surface of the magnet (**b**) scattering of Gd^3+^-Pdots reporters in the absence of magnets and (**c**) assembly of Gd^3+^-Pdots reporters along the surface of the magnet after a time period of 24 h.

In addition, the manipulation of Gd^3+^-Pdots reporters was tested by using a neodymium bar magnet. As observed from Figure 5b, the Gd^3+^-Pdots reporters are scattered in the solution, whereas they assemble and cover the surface of the magnet placed under the petri dish after a time period of 24 h (Figure 5c, also schematically represented in Figure 5a). This observation shows that dual-functional Gd^3+^-Pdots reporters could be manipulated by an external magnetic field and that the magnetic patterning and assembly on samples are feasible owing to their intrinsic magnetic properties. These results ascertain that the synthesized Gd^3+^-Pdots could serve as dual-functional fluoro-magnetic nanoprobes for bioimaging.

## 4. Conclusions

In conclusion, dual-functional Gd^3+^-Pdot reporters were successfully synthesized via a one-pot mini-emulsion technique for bioimaging applications. SEM, fluorescence microscopy, UV-visible and fluorescence spectroscopy, and magnetic levitation characterization results illustrated that the Gd^3+^-Pdot reporters possess fluorescence and magnetic properties, imparted by PTAA and Gd^3+^, respectively. Thus, the synthesized reporters could be utilized for multimodal bioimaging applications. Furthermore, with the post-functionalization of Gd^3+^, the Pdots could be magnetically manipulated via an external magnetic field. Apart from bioimaging, we foresee that the dual-functional Gd^3+^-Pdot reporters could be utilized as magnetic tweezers for manipulating micron sized objects.

## Figures and Tables

**Table 1:** Zeta potential measurements of Pdots and Gd^3+^ -Pdots.

Sample	Zeta Potential (mV)	Mobility (µmcm/Vs)	Conductivity (mS/cm)
Pdots	11.8 ± 0.60	0.922 ± 0.048	0.104
Gd^3+^-Pdots	6.10 ± 1.10	0.478 ± 0.086	0.106

## Data Availability

The raw/processed data required to reproduce these findings cannot be shared at this time as the data also forms part of an ongoing study.

## Supplementary Materials

The following supporting information can be downloaded at: https://www.mdpi.com/article/10.3390/nano12040642/s1, Figure S1: ^1^H NMR spectrum of poly (3-thiophene methyl acetate); Figure S2: FTIR-ATR spectrum of poly (3-thiophene methyl acetate) (PTMA) and polythiophene acetic acid (PTAA); Figure S3: Purification of Gd^3+^-Pdots from free Gadolinium.
