# Supplementary material for: Gadolinium and Polythiophene Functionalized Polyurea Polymer Dots as Fluoro-Magnetic Nanoprobes
Source: Nanomaterials (Basel). 2022 Feb 14;12(4):642. doi: 10.3390/nano12040642 (PMC8875818; doi:10.3390/nano12040642)
Supplement: Supplementary file 1 [file nanomaterials-12-00642-s001.zip › nanomaterials-1539577-supplementary.pdf]

nanomaterials

Supplementary Materials

Gadolinium and Polythiophene Functionalized Polyurea Polymer Dots as Fluoro-Magnetic Nanoprobes

Soner Karabacak [1], Alagappan Palaniappan [2], Tsang Siu Hon Tony [3], Teo Hang Tong Edwin [2,4], Balázs Gulyás [5,6,7], Parasuraman Padmanabhan [5,6,*] and Ümit Hakan Yildiz [1,8,9,*]

1 Department of Chemistry, İzmir Institute of Technology, Urla 35430, İzmir, Turkey; sonerkarabacak@iyte.edu.tr
2 School of Materials Science and Engineering, Nanyang Technological University, Singapore 639798, Singapore; alps@ntu.edu.sg (A.P.); e-e-HTTEO@ntu.edu.sg (T.S.H.T.)
3 Temasek Laboratories@NTU, 50 Nanyang Avenue, Singapore 639798, Singapore; SHTSANG@ntu.edu.sg
4 School of Electrical and Electronic Engineering, Nanyang Technological University, Singapore 639798, Singapore
5 Lee Kong Chian School of Medicine, Nanyang Technological University, Singapore 639798, Singapore; balazs.gulyas@ntu.edu.sg
6 Cognitive Neuroimaging Centre, Nanyang Technological University, 59 Nanyang Drive, Singapore 636921, Singapore
7 Department of Clinical Neuroscience, Karolinska Institute, 17176 Stockholm, Sweden
8 Department of Polymer Science and Engineering, İzmir Institute of Technology, Urla 35430, İzmir, Turkey
9 Denge Kimya, Velimese Industrial Region St. Ergene, 59860, Corlu/Tekirdag, Turkey
* Correspondence: ppadmanabhan@ntu.edu.sg (P.P.); hakanyildiz@iyte.edu.tr (Ü.H.Y.)

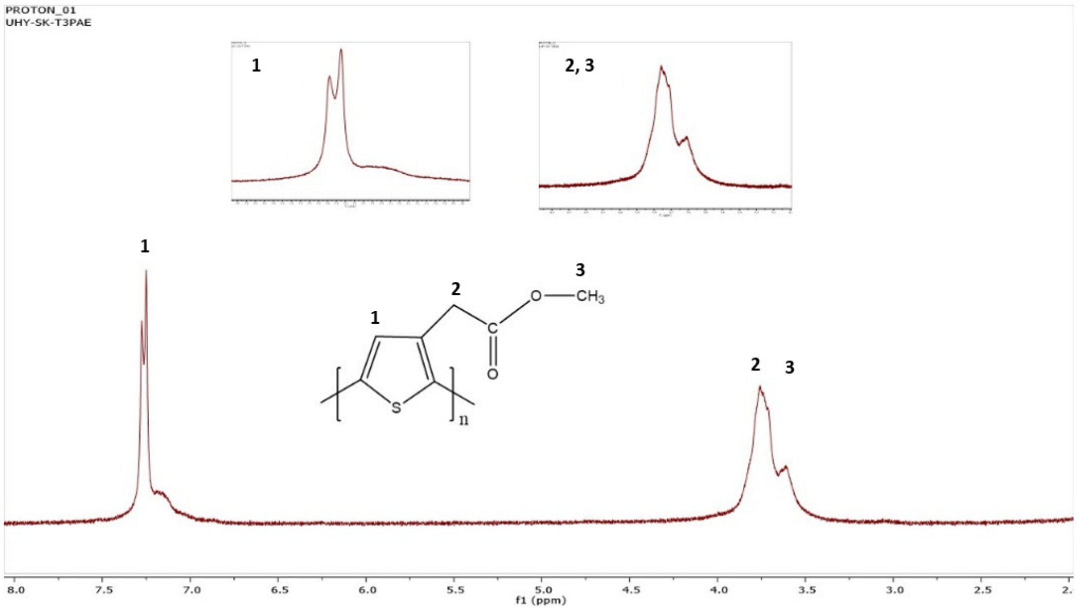

Figure S1. ^{1}H NMR spectrum of poly (3-thiophene methyl acetate).

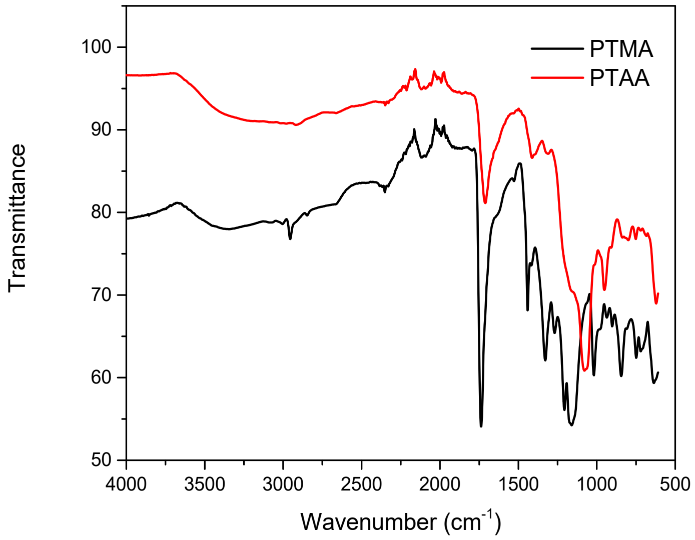

Figure S2. FTIR-ATR spectrum of poly (3-thiophene methyl acetate) (PTMA) and polythiophene acetic acid (PTAA).

We deduced the final yield based on the determination of unreacted gadolinium in Gd^{3+}-Pdots (Figure S3), Gd^{3+}-Pdots were dialyzed against a 0.05 M citrate solution (200 mL, pH:7.4) for five cycles (each cycle for 24 h) and free Gd^{3+} were analyzed by ICP-OES (inductively coupled plasma optic emission spectroscopy). The yield was calculated by the difference between Initial Gd^{3+} - Free Gd^{3+} = Bound Gd^{3+}. This calculation shows that the yield of Gd^{3+}-Pdots is 26.6%.

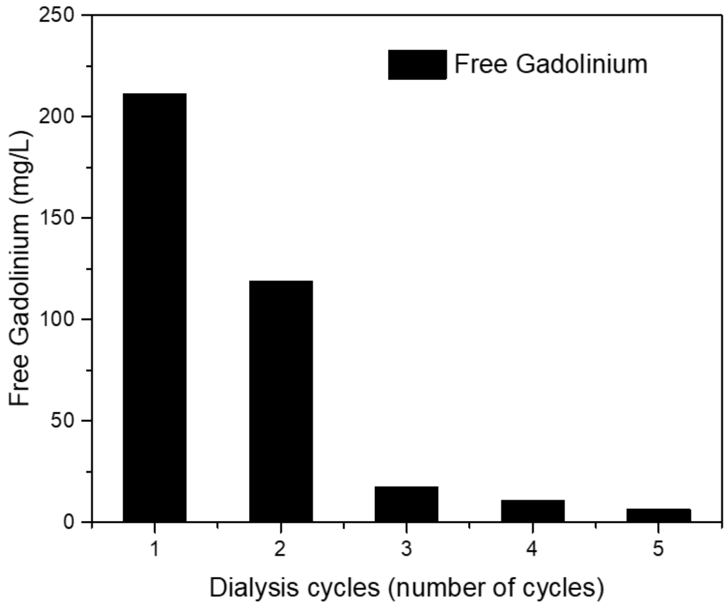

Figure S3. Purification of Gd^{3+}-Pdots from free Gadolinium.
